# A Preligamentous Variant of the Thenar Motor Branch of the Median Nerve

**DOI:** 10.5435/JAAOSGlobal-D-20-00029

**Published:** 2020-10-07

**Authors:** Edmund Patrick Mullin, J. Banks Deal, Kevin P. Krul

**Affiliations:** From F. Edward Hérbert School of Medicine, Uniformed Services University of the Health Sciences, Bethesda, MD (Mr. Mullin), and the U.S. Army Medical Service Corps, Tripler Army Medical Center, Honolulu, HI (Mr. Mullin, Dr. Deal, and Dr. Krul).

## Abstract

We report a rare variant of the thenar motor branch (TMB) of the median nerve. A preligamentous TMB was discovered during revision carpal tunnel release in a 49-year-old man. The prevalence and characteristics of TMB variations are discussed. The literature describing iatrogenic injury to the TMB variants, surgical treatment, and preoperative screening tools is reviewed. Recognition of anatomic variants of the TMB is essential considering notable consequences of iatrogenic injury.

We report a case of a rare preligamentous thenar motor branch (TMB) of the median nerve encountered during revision carpal tunnel release. Carpal tunnel syndrome (CTS) is an entrapment neuropathy in which the median nerve is compressed within the carpal canal. The goal of surgical management is to decompress the median nerve while avoiding iatrogenic injury. Current approaches to release the carpal tunnel include miniopen, endoscopic, and open techniques. The miniopen and endoscopic surgical dissection is an effort to reduce postoperative pain, and large series have validated their efficacy.^1^ However, the surgeon must be aware of the numerous anatomic variations of the TMB of the median nerve.^2^ When anatomic variants of the TMB are present, iatrogenic injury during carpal tunnel release may be avoided if prompt recognition is made by the surgeon.^3^

## Case Presentation

A 49-year-old man presented with recurrent right CTS after an endoscopic release three years before. He described complete relief for a period of 2 years, followed by gradual recurrence of numbness and paresthesia in his thumb, index, and long finger. The patient's main complaints were weakness and loss of dexterity. He was employed as a radiology technician.

On examination, the patient had mild atrophy of thenar muscles of his right hand and weakness with thumb opposition. Examination revealed a positive Phalen test, positive Durkan test, and positive Tinel test over the carpal tunnel but was negative for Tinel at the cubital tunnel. Sensation was grossly intact, although subjectively decreased over the median nerve distribution.

The patient desired to avoid surgery and attempted splinting without relief of symptoms. An injection of local anesthetic and corticosteroid provided temporary relief. Both electromyogram and MRI were obtained preoperatively; these demonstrated thickening of the median nerve at the carpal tunnel and denervation of the thenar musculature. He elected for revision of his carpal tunnel release using the open approach.

**Figure 1:**
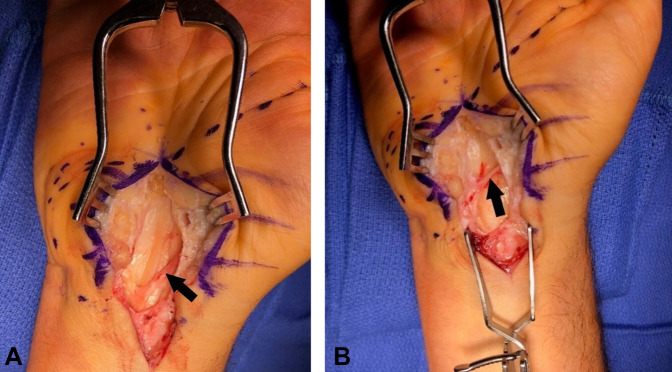
**A**, Intraoperative photograph demonstrating preligamentous thenar motor branch (TMB) of the median nerve before more extensive dissection. The black arrow points to the TMB of the median nerve. **B**, Intraoperative photograph of the preligamentous TMB of median nerve after further dissection. The black arrow points to the TMB of median nerve.

The procedure was done under general anesthesia in the operating room. The area overlying the carpal tunnel was injected with 1% lidocaine with epinephrine. The skin was incised in line with the fourth ray. Careful dissection under direct visualization was done through the subcutaneous fat and through the thickened palmar fascia. A preligamentous branch of the thenar motor nerve was discovered (Figure 1, A and B). The incision was extended proximally and distally to the point where the nerve was seen diving into the thenar musculature.

After extending the incision proximally in a Brunner fashion, the palmar fascia and antebrachial fascia were released. Neurolysis of the motor branch was done because portions of the nerve were compressed in scar tissue. Once the TMB could be safely protected, the transverse carpal ligament (TCL) was released. Direct visualization and palpation of the carpal canal demonstrated a full and complete release. The tourniquet was released, hemostasis was established, the wound was irrigated, and the skin was closed. The wound was dressed, and a short arm splint was applied. Given the dissection required, postoperative immobilization was maintained for two weeks. At the one-month follow-up, the patient denied any motor complaints with return of normal function.

## Discussion

Anatomical variants of the TMB are quite prevalent, although infrequently encountered in clinical practice. Typically, the TMB emerges from the median nerve becoming the recurrent motor branch at the distal margin of the TCL before entering the thenar muscles.^2^ Nearly every TMB arises from the radial or palmar-radial aspect of the median nerve (98%).^4^ Poisel was the first to classify three variant types of branching of the TMB: extraligamentous, subligamentous, and transligamentous.^4^ Lanz^2^ expanded on Poisel's classification, establishing four branching patterns of distal portion of the median nerve. Type 1 describes a solitary TMB emerging within or distal to the carpal tunnel. Type 2 describes accessory TMBs distal to the tunnel. Type 3 is a high division of the median nerve, with the TMB arising from one of divided branches at the level of the tunnel. Finally, type 4 describes accessory TMBs proximal to the carpal tunnel.

**Figure 2:**
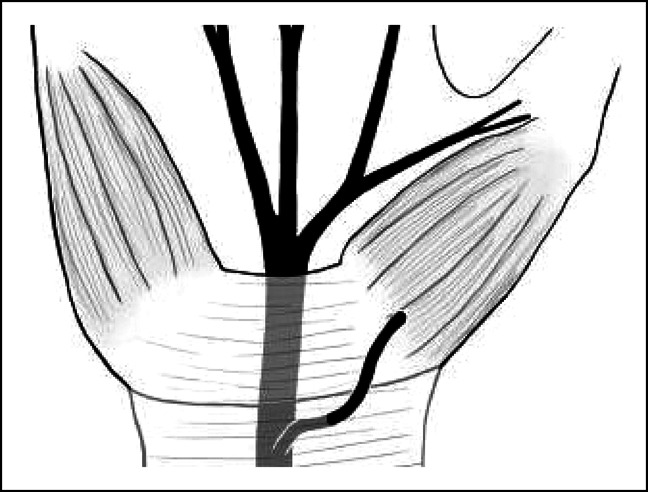
Illustration of a preligamentous course of the thenar motor branch of the median nerve adapted with permission from Dr. Krzysztof Tomaszeski.^4^

Lanz's system describes some, although not all, of the possible variations in anatomy encountered. Our patient's TMB arose from the median nerve proximal to the carpal tunnel, then passed superficial to the TCL before entering the thenar musculature. No anastomosis with a late-branching nerve was encountered. This preligamentous variant shares characteristics with both Lanz Types 1 and 3, although the classification does not neatly apply (Figure 2). The surgeon must bear in mind that individual patients' anatomies do not always fit the textbook pattern. In a metanalysis including 3,918 cases, Henry et al^4^ calculated the pooled prevalence of each Lanz Type. Given the rarity of a preligamentous course of the TMB, its prevalence was not included in the meta-analysis.

Although the landmark study of Green and Morgan^5^ did not explicitly mention a preligamentous course of the TMB, they noted that 93% of subjects with muscle fibers superficial to or interposed within the TCL displaced anomalous TMBs. Henry et al^4^ further described this correlation between hypertrophic muscle overlying the TCL and the transligamentous variant of the TMB. This Lanz type 1 variant was more commonly found in the hands of hypertrophic thenar muscles (23.4%) compared with those without hypertrophic musculature (1.7%). The source of the muscle that overlies the TCL is still a matter of dispute.

Among 272 subjects whose carpal tunnels were examined bilaterally, Green and Morgan^5^ noted that less than one-third of subjects had a purely unilateral anomalous TMB. More recently, Jegal et al^6^ assessed 192 open carpal tunnel releases, finding 25 wrists with muscle overlying the TCL—four of which were bilateral cases. Fourteen of the 25 were found to have an ulnar course of the TMB that tracked into this hypertrophic muscle overlying the TCL. Given the rarity of TMB variants arising from the ulnar side, in conjunction with the fact that one in 10 patients have a transligamentous course of the TMB, Henry et al supports Lanz's recommendation for an ulnar surgical approach with careful dissection of the carpal tunnel.^2,4^

The use of ultrasonography as a preoperative screening tool to identify anomalous neural anatomy before carpal tunnel release has been reported. Petrover et al found that high-frequency ultrasonography could identify anatomic variations of the TMB in the carpal tunnel. In a series of 15 fresh cadavers, their group assessed for anomalous hypertrophic muscle and the orientation of the TMB using an 18-MHz ultrasonography. Then, dissection was carried out to determine accuracy of the ultrasonography examination. They found that 100% of anomalies were appropriately detected by ultrasonography examination for type 1 and type 3 variants.^7^ Furthermore, Petrover discovered that some people had more than one TMB branch, supporting Kozin's^8^ report that 4% of the population had more than one motor branch. The low cost of ultrasonography compared with the high cost for iatrogenic injury to the TMB—even when clinical suspicion is low—may warrant implementation of this procedure in select cases.

The selection of the surgical technique must also be considered. Lutsky et al analyzed the rate of TMB variants during endoscopic or miniopen carpal tunnel releases. The prospective, multisurgeon study found anomalous TMB variants at a rate of less than 0.5%. This was attributed to the small surgical exposure and the location of the dissection along the ulnar aspect of the TCL.^9^

Anomalous TMB variants may increase the risk of iatrogenic injury during carpal tunnel release. Although CTS and anatomic variations of the TMB had been previously documented, Lilly et al were the first to report cases of iatrogenic injury to the TMB during carpal tunnel release. Furthermore, Lilly and Magnell^3^ felt that his cases also presented the first results of delayed repair of such an iatrogenic injury in the literature. The sequelae of iatrogenic injury may be substantial, especially in the setting of well-functioning or recoverable thenar musculature. In the setting of irrecoverable thenar muscle function after prolonged denervation or when adequate function is obtained in the setting of hypertrophy of the deep head of the flexor pollicis brevis (ulnar nerve), iatrogenic injury to the TMB may be less consequential. Current surgical treatments for iatrogenic nerve injury include primary end-to-end repair and reconstruction using nerve collagen conduits, autograft, or decellularized nerve allografts. Superior outcomes are reported when tension-free end-to-end repair is achieved.^10^

## Conclusion

We reported a rare case of a patient with a preligamentous TMB arising proximal to the carpal tunnel. Our patient had recurrent CTS with predominantly motor symptoms due to scarring around his preligamentous TMB. Open revision surgery allowed for identification and neurolysis of this anomalous branch, rapidly improving the patient's symptoms. It is likely that simply rereleasing the TCL would not have helped his motor symptoms. The importance of recognizing aberrant anatomy cannot be overstated. Strategies to avoid injury include recognition of hypertrophic muscle overlying the TCL and careful dissection. Finally, ultrasonography may be of use as a screening test to identify TMB anomalies preoperatively.
